# Isolated Gastric Variceal Hemorrhage Secondary to Idiopathic Sinistral Portal Hypertension

**DOI:** 10.7759/cureus.16165

**Published:** 2021-07-04

**Authors:** Mona Abraham, Shreyans Doshi, Mohammad Maysara Asfari, John Erikson L Yap, H. Gregory Bowers

**Affiliations:** 1 Gastroenterology and Hepatology, Augusta University Medical College of Georgia, Augusta, USA; 2 Interventional Radiology, Augusta University Medical College of Georgia, Augusta, USA

**Keywords:** sph, sinistral portal hypertension, gastric variceal hemorrhage, gi bleed, splenic artery embolization, splenic vein thrombosis, upper gi bleed, isolated gastric varices, ugib, left-sided portal hypertension

## Abstract

Sinistral portal hypertension (SPH), also known as left-sided portal hypertension or segmental portal hypertension, is a rare cause of upper gastrointestinal bleeding. Historically, SPH is a result of obstruction of the splenic vein often secondary to pancreatic pathology. To our knowledge, there are no reported cases of idiopathic SPH in which the findings cannot be attributed to any etiology. It is important to do a detailed workup to rule out common pathologies of SPH before making a diagnosis of idiopathic SPH. Treatment of gastric variceal bleed secondary to idiopathic SPH can be challenging and requires a multidisciplinary approach with surgery and interventional radiology. Our patient’s history, examination findings, and imaging revealed no identifiable cause for SPH suggesting idiopathic SPH. We describe a case of isolated gastric variceal hemorrhage due to idiopathic SPH that was successfully treated.

## Introduction

Sinistral portal hypertension (SPH) is defined as elevated pressure confined to the gastrosplenic side of the portal venous system. SPH differs from other forms of portal hypertension in that the liver function is preserved and a patent portal vein is present. It is most commonly secondary to pancreatitis, malignancy, pancreatic pseudocysts, benign pancreatic neoplasms, splenic vein thrombosis, and retroperitoneal fibrosis [1]. SPH can be diagnosed by a combination of endoscopy, liver function tests, ultrasound with Doppler, and contrast-enhanced computed tomography scan or magnetic resonance imaging of the abdomen. Portal pressure and hepatic wedge pressure measurement would be essential to differentiate SPH from right-sided portal hypertension in cases without identifiable etiology for SPH. Although most cases of SPH are secondary to associated pathology, none have been reported of patients with idiopathic SPH. Our patient was unique in that he had isolated gastric varices without any identifiable cause. Here, we present a rare case of isolated gastric variceal hemorrhage secondary to idiopathic SPH which was successfully managed by splenic artery embolization.

This article was previously presented at the American College of Gastroenterology Annual Scientific Meeting held in October 2020.

## Case presentation

**Figure 1:**
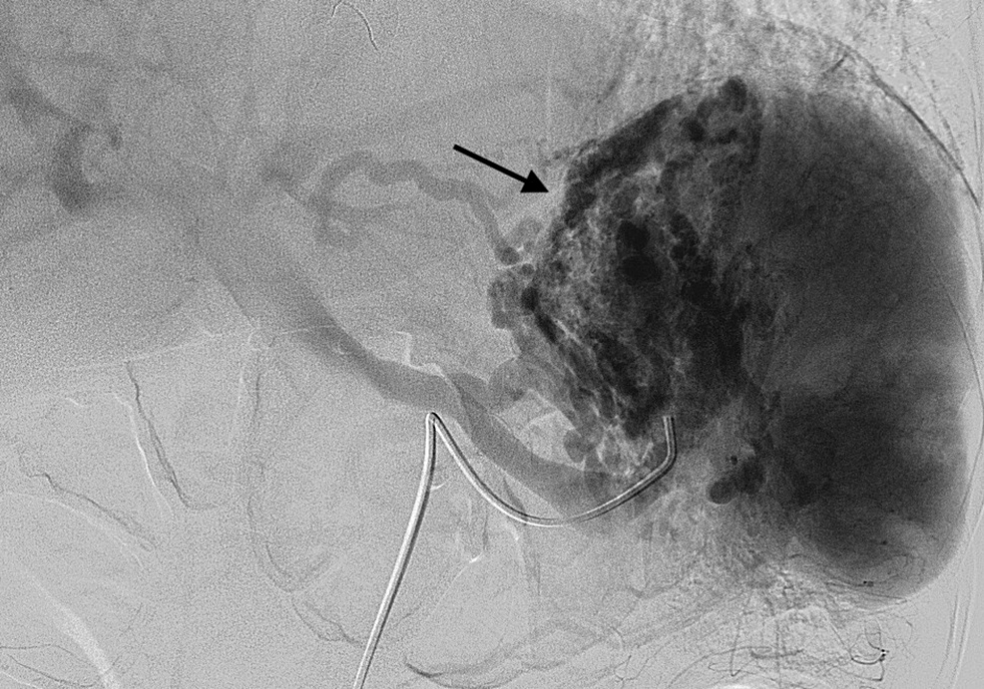
Splenic arteriogram prior to embolization demonstrating gastric varices.

**Figure 2:**
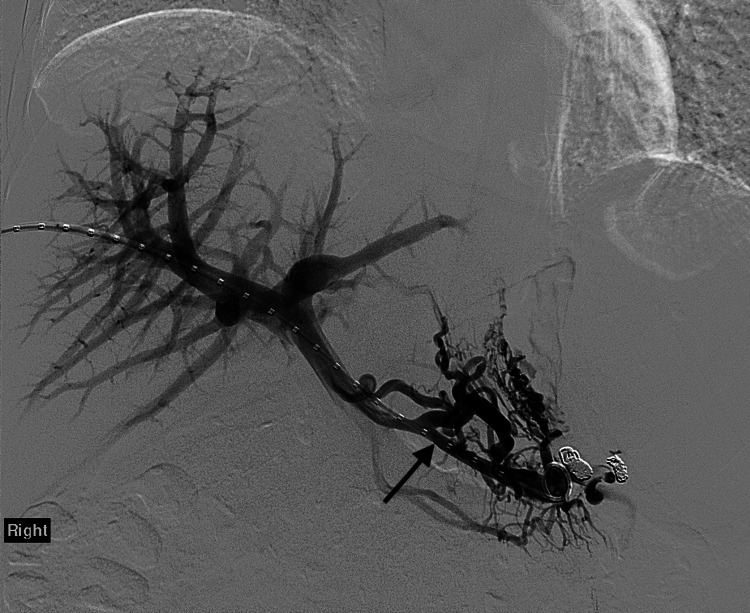
Splenic vein and portal venogram after splenic artery embolization.

**Figure 3:**
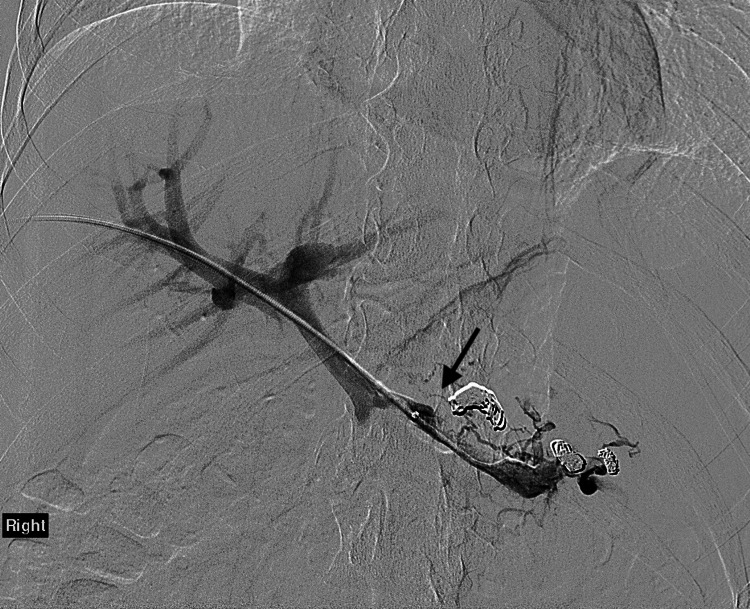
Splenic vein and portal venogram after embolization of gastric varices.

A 53-year-old African American male with a history of developmental delay, schizophrenia, and paranoia underwent an emergent exploratory laparotomy and small bowel resection followed by a primary end-to-end anastomosis due to multiple self-inflicted stab wounds. No varices were seen in the abdomen during the exploratory laparotomy and the liver appeared normal as well. On postoperative day 10, he developed melena and epigastric pain, and his hemoglobin reduced from 10 g/dL to 7 g/dL. A repeat exploratory laparotomy and computed tomography angiogram (CTA) did not find the etiology for the bleeding. Esophagogastroduodenoscopy (EGD) was performed and showed a large isolated gastric varix (IGV-1) in the fundus and no esophageal varices. The IGV-1 had an area with active oozing. He was then emergently sent for an angiogram for possible embolization. On interventional radiology (IR) evaluation, it was noted that the portal vein and the splenic vein were widely patent, and his portal pressures were normal with a 4 mmHg hepatic venous pressure gradient, yet there were multiple large gastric varices (Figure 1) without active bleeding. The following day splenic artery embolization (SAE) was successfully performed by IR to achieve hemostasis. Following SAE, he had no further melena and his hemoglobin remained stable. An extensive review of his history and imaging ruled out pancreatitis, malignancy, cirrhosis, or any instrumentation-related injury of the vasculature. The patient did not have any previous history of GI bleeding as well. Repeat CTA and EGD were done following SAE which confirmed decreased caliber of the gastric varices. Given the persistence of the gastric varices, albeit smaller in caliber, he underwent percutaneous ultrasound-guided transhepatic portography by IR four weeks after the initial presentation (Figure 2). Direct portal venous pressure was 16 mmHg which correlated with the prior measurements of right atrial pressure and free hepatic venous pressure (17 mmHg), confirming there was no portosystemic gradient or right-sided portal hypertension. Gastric varices were successfully treated with coil embolization (Figure 3). The patient’s hemoglobin remains stable at 12.9 g/dL at outpatient follow-up without evidence of melena and complete resolution of varices.

## Discussion

SPH is a localized form of portal hypertension sometimes referred to as left-sided portal hypertension, segmental, regional, compartmental, lineal, or splenoportal hypertension [2]. Despite the terminology, SPH exhibits normal portal pressure and portal pressure gradient, differentiating it from right-sided portal hypertension. Although SPH makes up less than 1% of cases of upper gastrointestinal bleeding (UGIB), it can lead to potentially life-threatening UGIB [3]. It is most associated with pancreatic pathology, including pancreatic malignancy, pancreatic pseudocysts, and chronic pancreatitis. Due to the anatomical proximity of the pancreas to the splenic vein, these pathologies can lead to splenic vein compression causing backpressure into the left portal venous system resulting in reversal of venous flow and isolated gastric varices. In addition, the proximity of the pancreas to the splenic vein puts patients at risk for splenic vein thrombosis (SVT) [3]. Like SVT, splenic vein occlusion secondary to organs, masses, edema, enlarged lymph nodes, and splenic artery aneurysm can also lead to SPH [2]. Although these etiologies have been discussed in the literature, we present a unique case of idiopathic SPH. In our patient, CTA confirmed patent splenic and portal veins. Further imaging and history ruled out pancreatic pathology, malignancy, and liver pathologies. We suspect that this could have been the initial variceal bleeding of the patient from SPH.

Few case reports have demonstrated isolated gastric varices in the absence of SVT [4]. Unlike patients with right-sided portal hypertension, patients with SPH more commonly bleed from gastric varices, and UGIB is often the presenting symptom as the majority are asymptomatic [2]. Although our patient lacked splenomegaly, SPH should be considered in any patient with isolated gastric varices, splenomegaly, and normal liver function [2].

Our patient’s bleeding was controlled with SAE which was used to block direct arterial inflow to the spleen, and thereby reduce the outflow venous pressure that results in gastric varices [5]. Alternative therapies include endovascular stents, splenectomy, and vascular surgery for patients who are good surgical candidates [6,7].

## Conclusions

SPH is a rare but life-threatening cause of UGIB that should be suspected in any patient with isolated gastric varices and normal portal pressure. Although most commonly secondary to pancreatic pathologies requiring thorough workup, it can be idiopathic. Further studies are needed to conclude if there is a congenital component to SPH that may be a contributing factor to idiopathic SPH.
